# Incidental Wolff-Parkinson-White Syndrome Discovered Following Dicyclomine Use: A Case Report

**DOI:** 10.5811/cpcem.53127

**Published:** 2026-05-23

**Authors:** John Wahhab, Natalie Loveridge, Manar Siaj

**Affiliations:** * Holy Cross Hospital, Department of Emergency Medicine, Chicago, Illinois; † Chicago Medical School at Rosalind Franklin University of Medicine and Science, Department of Emergency Medicine, North Chicago, Illinois

**Keywords:** Wolff-Parkinson-White, dicyclomine, arrhythmias, case report

## Abstract

**Introduction:**

Wolff-Parkinson-White syndrome is a congenital conduction disorder involving an accessory pathway that predisposes patients to reentrant tachyarrhythmias and, in rare cases, sudden cardiac death. While often asymptomatic, it may predispose patients to serious tachyarrhythmias, particularly under conditions that enhance atrioventricular (AV) conduction. Risk stratification using noninvasive and invasive tools such as electrophysiologic studies is critical to identifying high-risk individuals and guiding treatment decisions such as catheter ablation. Pharmacologic agents that alter autonomic tone may unmask latent pre-excitation. Dicyclomine, an anticholinergic agent used for gastrointestinal disorders, is not an AV-nodal blocking drug but exerts vagolytic effects that can increase sinus rate and AV nodal conduction. Dicyclomine’s vagolytic effects and potential for interaction with other proarrhythmic drugs warrant caution in patients with conduction abnormalities, structural heart disease, or autonomic sensitivity.

**Case Report:**

We report a case of a 36-year-old male who presented to the emergency department with abdominal symptoms and was incidentally found to have Wolff-Parkinson-White pattern following administration of intramuscular dicyclomine.

**Conclusion:**

This case highlights the potential importance of cardiac monitoring, even in patients with non-cardiac chief complaints, particularly when anticholinergic agents are administered.

## INTRODUCTION

Wolff-Parkinson-White syndrome is a congenital cardiac conduction disorder characterized by the presence of an accessory pathway between the atria and ventricles, predisposing individuals to reentrant tachyarrhythmias and, in rare cases, sudden cardiac death.^1^,^2^ While many individuals with the disorder are asymptomatic, even silent or intermittent pre-excitation patterns carry the potential for life-threatening arrhythmias, particularly under conditions that stimulate sinoatrial node activity and impede atrioventricular (AV) conduction.^3^,^4^ Risk stratification through both invasive and noninvasive modalities, such as electrophysiologic studies, remains essential in identifying high-risk patients and guiding definitive therapies like catheter ablation.^5^,^6^

Despite improved diagnostic tools and curative interventions, the intersection between pharmacologic agents and pre-existing conduction disorders remains an under-recognized hazard in clinical practice. Dicyclomine, a commonly prescribed anticholinergic agent used to treat gastrointestinal motility disorders, may pose a unique risk. While not an AV-nodal blocking agent and not classically associated with malignant arrhythmias, dicyclomine exerts vagolytic effects that increase sinoatrial node activity automaticity and AV nodal conduction.^7^ These autonomic effects raise a theoretical concern that anticholinergic medications could unmask latent ventricular pre-excitation, particularly in populations with structural heart disease, conduction abnormalities, or autonomic sensitivity.^8^,^9^,^10^

This case report describes the incidental identification of a Wolff-Parkinson-White pattern following intramuscular dicyclomine administration. The novelty of this case lies not in the diagnosis of Wolff-Parkinson-White itself but in the temporal association between anticholinergic exposure and the unmasking of pre-excitation. This report serves as a hypothesis-generating observation rather than evidence of a causal relationship.

## CASE REPORT

A 36-year-old male with no previous medical history presented to the emergency department with a five-day history of cramping periumbilical abdominal pain and diarrhea. The patient reported minimal relief with over-the-counter bismuth subsalicylate. He denied fever, chills, nausea, vomiting, lightheadedness, dizziness, weakness, or shortness of breath. On initial evaluation, his vital signs were as follows: heart rate, 90 beats per minute; respiratory rate, 16 breaths per minute; blood pressure, 145/88 millimeters of mercury; oxygen saturation, 98% on room air; and temperature, 98.3 °Fahrenheit. Physical examination revealed a soft abdomen with minimal periumbilical tenderness.

A peripheral intravenous line was established, and the patient was placed on continuous cardiac monitoring and pulse oximetry while awaiting physician evaluation. Initial rhythm on the cardiac monitor showed normal sinus rhythm. The complete blood count was within normal limits, showing no leukocytosis. The complete metabolic panel showed normal electrolytes, including a potassium level of 4.3 milliequivalents per liter (mEq/L) (reference range: 3.5–5.0 mEq/L) and a sodium level of 135 mEq/L (135–145 mEq/L). The serum magnesium level was also normal at 2.0 mEq/L (1.5–2.4 mEq/L).

The patient received 20 milligrams of intramuscular dicyclomine. Following administration, a change in cardiac rhythm was observed on the monitor. A 12-lead electrocardiogram (ECG) was performed and demonstrated delta waves concerning for Wolff-Parkinson-White pattern (Image). A high-sensitivity cardiac troponin was then obtained, measuring 8 picograms per milliliter (pg/mL). A repeat troponin two hours later was 9 pg/mL (< 20 pg/mL for men). Both values were interpreted as negative per institutional assay standards.

Repeat clinical assessment revealed no development of cardiovascular symptoms, including chest pain, palpitations, shortness of breath, lightheadedness, or dizziness. The patient remained hemodynamically stable. He reported improvement in his abdominal symptoms following dicyclomine administration and was able to tolerate oral intake without recurrence of pain or diarrhea. Electrophysiology consultation was obtained and outpatient evaluation was recommended. Unfortunately, the patient was lost to follow-up.

*CPC-EM Capsule*

What do we already know about this clinical entity?

*Medications contraindicated in Wolff-Parkinson-White are atrioventricular (AV) nodal blocking agents, which increase the risk of arrhythmias.*

What makes this presentation of disease reportable?

*We report the incidental identification of a Wolff-Parkinson-White pattern following intramuscular dicyclomine administration.*

What is the major learning point?

*Anticholinergic agents may unmask latent ventricular pre-excitation through vagolytic enhancement of sinoatrial node activity and AV node conduction.*

How might this improve emergency medicine practice?

*Clinician should be aware of how autonomic modulation may reveal latent conduction abnormalities during routine care.*

## DISCUSSION

This case highlights the incidental identification of Wolff-Parkinson-White pattern in a patient presenting with gastrointestinal complaints following intramuscular administration of dicyclomine. The significance of this report lies in the temporal association between anticholinergic exposure and unmasking of ventricular pre-excitation, rather than diagnosis of the disorder itself. Wolff-Parkinson-White pattern is often identified on ECG by the presence of delta waves and a shortened PR interval, reflecting early presenting premature ventricular depolarization via an accessory pathway.^2^ Medications traditionally contraindicated in Wolff-Parkinson-White, such as adenosine, verapamil, diltiazem, digoxin, intravenous amiodarone, and beta-blockers, are AV nodal blocking agents that can promote conduction over the accessory pathway, increasing the risk of rapid ventricular rates and potentially life-threatening arrhythmias.^11^

In contrast, dicyclomine is not known to slow AV nodal conduction or directly facilitate accessory pathway activation. Instead, it competitively inhibits acetylcholine at muscarinic receptors, reducing vagal tone and thereby stimulating sinoatrial node activity and enhancing AV nodal conduction.^12^ While this autonomic effect provides a biologically plausible mechanism by which preexcitation may become manifest, this observation does not establish causality. No prior cases directly linking dicyclomine to unmasking of Wolff-Parkinson-White have been described in literature. Accordingly, this case should be interpreted as hypothesis-generating rather than evidence of a drug-induced conduction abnormality.

Recognition of incidental Wolff-Parkinson-White pattern remains clinically relevant, as risk stratification through electrophysiologic studies may be warranted in high-risk patients. High-risk features include a short pre-excited RR interval during induced or spontaneous atrial fibrillation ≤ 250 milliseconds (msec) indicating rapid conduction capability of the accessory pathway, accessory pathway effective refractory period ≤ 240–250 msec, multiple accessory pathways, and a history of syncope, male sex, and age < 30 years.^13^–^15^ However, this case report does not support changes in prescribing practices or routine cardiac screening prior to anticholinergic administration. Rather, it emphasizes clinician awareness of how autonomic modulation may reveal latent conduction abnormalities during routine care.

One limitation of this case report is that the observed relationship between intramuscular dicyclomine administration and the identification of ventricular pre-excitation represents a temporal association only; causality cannot be established from a single observational case. Second, the patient did not develop any documented arrhythmias or cardiac symptoms during the clinical encounter, limiting conclusions regarding clinical risk or arrhythmogenic potential. Third, no ECG was obtained prior to the administration of dicyclomine, so there was no formal ECG for comparison. Fourth, no electrophysiology study or advance cardiac testing was performed on the patient, precluding definitive characterization of accessory pathway properties and risk stratification. Finally, the patient was lost to follow-up, which prevented the assessment of long-term outcomes.

## CONCLUSION

This case report describes a temporal association between intramuscular dicyclomine administration and incidental identification of a Wolff-Parkinson-White pattern. The novelty lies in the proposed autonomic mechanism: vagolytic enhancement of sinoatrial node activity and atrioventricular node conduction, in which anticholinergic agents may unmask latent ventricular pre-excitation. While causality cannot be established, this observation serves to generate hypotheses and highlights the importance of cautious interpretation of incidental ECG findings in the context of pharmacologic autonomic modulation.

## Figures and Tables

**Image.**
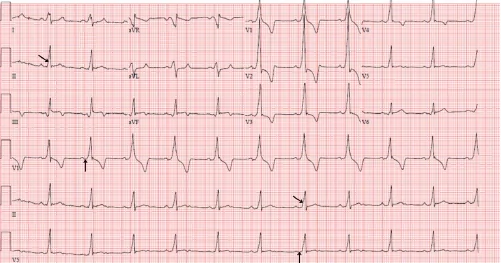
Electrocardiogram (ECG) revealing Wolff-Parkinson-White pattern in a patient, following the administration of intramuscular dicyclomine. Arrows indicate delta waves and shortened PR-intervals, which are key ECG findings indicating Wolff-Parkinson-White syndrome.
